# Local cold adaption increases the thermal window of temperate mussels in the Arctic

**DOI:** 10.1093/conphys/coz098

**Published:** 2019-12-23

**Authors:** J Thyrring, R Tremblay, M K Sejr

**Affiliations:** 1 British Antarctic Survey, High Cross, Madingley Road, CB3 0ET, Cambridge, United Kingdom; 2 Department of Zoology, University of British Columbia, 4200 - 6270 University Blvd., V6T 1Z4, Vancouver, British Columbia, Canada; 3 Homerton College, Hills Road, CB2 8PH, Cambridge, United Kingdom; 4 Institut des sciences de la mer, Université du Québec à Rimouski, G5L 3A Rimouski, Canada; 5 Arctic Research Centre, Department of Bioscience, Aarhus University, 8000 Aarhus C, Denmark

**Keywords:** climate change, distribution model, mytilus, plasticity, range shifts, temperature, thermal tolerance

## Abstract

Species expand towards higher latitudes in response to climate warming, but the pace of this expansion is related to the physiological capacity to resist cold stress. However, few studies exist that have quantified the level of inter-population local adaptation in marine species freeze tolerance, especially in the Arctic. We investigated the importance of cold adaptation and thermal window width towards high latitudes from the temperate to the Arctic region. We measured upper and lower lethal air temperatures (i.e. LT and LT_50_) in temperate and Arctic populations of blue mussels (*Mytilus edulis*), and analysed weather data and membrane fatty acid compositions, following emersion simulations. Both populations had similar upper LT (~38 °C), but Arctic mussels survived 4°C colder air temperatures than temperate mussels (−13 vs. −9°C, respectively), corresponding to an 8% increase in their thermal window. There were strong latitudinal relationships between thermal window width and local air temperatures, indicating Arctic mussels are highly adapted to the Arctic environment where the seasonal temperature span exceeds 60°C. Local adaptation and local habitat heterogeneity thus allow leading-edge *M. edulis* to inhabit high Arctic intertidal zones. This intraspecific pattern provides insight into the importance of accounting for cold adaptation in climate change, conservation and biogeographic studies.

## Teaser

Climate warming redistributes species towards the Arctic, but current knowledge on how non-arctic species can adapt to the Arctic is very limited. This study shows blue mussels are able to adjust their thermal physiology to the extreme cold of the Arctic, hereby expanding further north. This new information on infra-specific adaptation is important for conservation in a climate change era.

## Introduction

In response to global warming, species are redistributing in a poleward direction. Thus, a fundamental task is to understand the impacts of climate change on species distribution and the associated effects on ecosystem functioning. The use of correlative species distribution modelling, which is based on thermal windows, has been widely applied to predict future distributional changes (Elith and Leathwick, 2009; Bennett *et al*., 2019). The thermal window is the temperature range between an organism’s upper and lower lethal temperature limits (LT_max_ and LT_min_), defined as the temperature at which individuals perish, and is the temperature range an organism can physiologically tolerate and survive. For numerous species, the thermal window is merely assessed in parts of their distribution; hence, the majority of models are based on limited data, and consequently assume conspecific populations share a common thermal tolerance, regardless of the local climatic conditions (Bennett *et al*., 2019). This approach implies that edge populations (equatorward and poleward) are more sensitive to temperature abnormalities because they live closer to their lethal limits. However, widely distributed species living in heterogeneous thermal regimes often adapt their thermal tolerance (e.g. *via* local adaptation or phenotypic acclimation) in response to local temperatures throughout their distributional range (Willett, 2010; Sanford and Kelly, 2011; Chen *et al*., 2018). Thus, adaptation improves species fitness and resilience to climate change, and including adaptation in climate change research is a necessity to understand how warming affects species distribution (Bush *et al*., 2016).

Local adaptation in upper thermal limits has been demonstrated in a range of marine organisms (reviewed by Sanford and Kelly, 2011), but studies comparing intraspecific local adaptation are generally rare, especially with regard to the lower thermal limit (local cold adaptation) and changes in thermal window width. The capacity to adapt to cold conditions is important for organism’s distribution, and for our capability to predict future range shifts. For example, temperate intertidal species that expand into the Arctic are exposed to extreme sub-zero temperatures during winter low tides (Thyrring *et al*., 2017a). Thus, sub-zero temperatures act as a barrier for poleward range shifts because exposure to winter air temperatures can offset the redistribution facilitated by the long-term atmospheric warming (Wethey *et al*., 2011). However, despite the obvious importance of cold adaptation, inter-population differences in lower thermal tolerance remains understudied (see Howells *et al*., 2013; Dennis *et al*., 2014; Wallace *et al*., 2014).

The blue mussel *Mytilus edulis*, and *Mytilus trossulus* and *Mytilus galloprovincialis* all belong to the cryptic ‘*M. edulis* species complex’, and ﻿are morphologically similar but physiologically distinct (Fly and Hilbish 2013; Tremblay and Landry 2016; Thyrring *et al*., 2017b). Pure populations of *M. edulis* are rare in the northern hemisphere, where extensive hybrid zones are found throughout Europe, North America and the Arctic (Mathiesen *et al*., 2017). To demonstrate the importance of cold adaptation on species distribution, we investigated lower thermal limits in relation to local air temperature conditions in *M. edulis* collected from temperate Denmark and Arctic Greenland, which represents the northernmost pure *M. edulis* population (Mathiesen *et al*., 2017). We further studied the upper thermal limit because previous work has suggested the thermal window shift downwards in adjustment with latitude. This means that populations at high latitudes have a suppressed upper thermal limit compared to populations at lower latitudes (Willett, 2010). Suppressed upper thermal limits can affect high-latitude populations, as extreme high summer temperatures, and not only temperature averages, are important for biogeographic patterns (Seabra *et al*., 2015).

Finally, we investigated a mechanistic process behind cold adaptation by testing if a relationship between thermal limits and cell membrane fatty acid composition exists. We did so because compositional change of the membranes fatty acids, through a process termed homeoviscous adaptation, has been emphasised as a general adaptive trait to survive sub-zero temperatures (Hazel and Williams, 1990; Hazel, 1995). Normal functioning membranes exists in a liquid-crystalline phase, but when cooled sufficiently, they transits to an ordered gel phase, whereby becoming partly dis-functional, losing selective properties and leak cell content (Hazel, 1995). To avoid lethal membrane damage at low temperatures, ectotherms increases the degree of unsaturation resulting in a more disordered membrane, which is less likely to undergo phase transition (Hazel and Williams, 1990; Pernet *et al*., 2007a). For instance, in the marine environment, the oyster *Crassostrea virginica* show fatty acid compositional changes in gills membrane cells in response to daily temperature fluctuations (Pernet *et al*., 2007a). However, the importance of homeoviscous adaptation in response to air temperature variation during emersion in geographically separated marine invertebrates remains poorly understood.

## Materials and methods

### Air temperature

**Figure 1:**
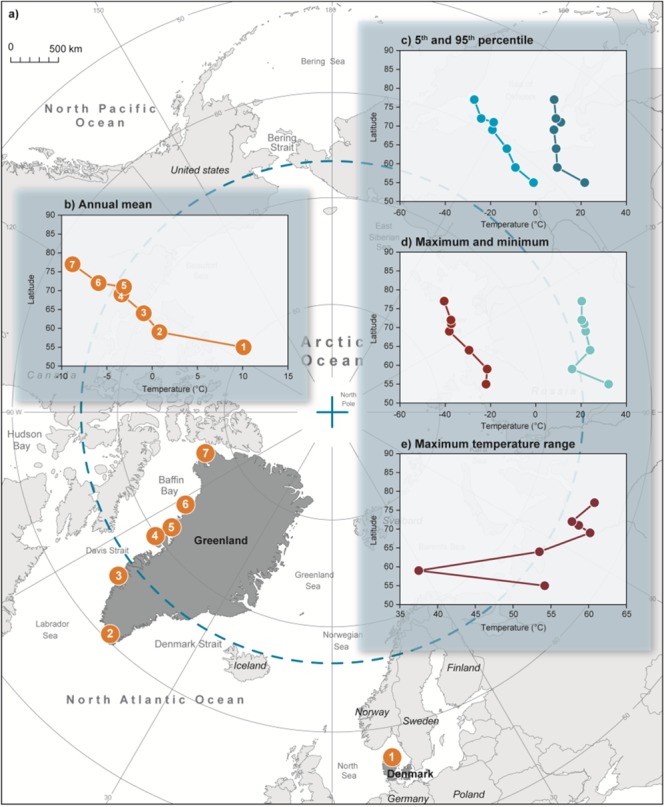
(a) Map of weather stations across 32° of latitude from Denmark (location 1) to Greenland (locations 2–6). Patterns in: (b) annual mean, (c) fifth (light blue) and 95th (blue) percentile, (d) minimum (red) and maximum (teal) and (e) maximum temperature range. Mussels were collected at locations 1 and 3.

Daily air temperatures were collected from seven local weather stations; One in Denmark (station no. 6132) and six along the south and west Greenland coast from Angissoq (station no. 4285); Nuuk (station no. 4250); Disko Island (station no. 4220/4224); Uummannaq (station no. 4212/4213); Upernavik (station no. 4209/4210/4211) to Qaanaaq (station no. 4201/4205), spanning 32° of latitude (Fig. 1a). We calculated local annual means, fifth percentile, 95th percentile, winter minimum and summer maximum by using all available temperature measurements from the period 1958 to 2016. Finally, we calculated the maximum air temperature range as the difference between the minimum and maximum temperature measured at the weather stations. The data were provided by the Danish Meteorological Institute; www.research.dmi.dk (Cappelen 2017a, b).

### Animals and holding conditions

This experiment was conducted on intertidal mussels (size 36.9 ± 4 mm) collected at similar shore heights in Arctic Greenland (Kobbefjord, 64°N) and Denmark (the Wadden Sea, 55°N) in August 2015. The Greenland population is the northernmost confirmed population of pure intertidal *M. edulis* (Mathiesen *et al*., 2017), and Wadden Sea mussels represent a pure temperate population (Kijewski *et al*., 2019). Individuals collected in Greenland were kept in aerated tanks before transported on ice to laboratory facilities at the Kattegatcentret aquarium, Denmark, within 48 hours of collection. Danish individuals were immediately placed on ice and transported to the aquarium within 3 hours of collection. All mussels were kept in open flow through 10°C seawater (salinity 27), and acclimated under identical laboratory condition for 5 months. The prolonged acclimation was used to eliminate any differences in physiology, which were due to their recent environmental history (Sokolova and Pörtner 2001). This approach allows for an analysis of irreversible (likely genetic) physiological differences amongst the two populations (Sanford and Kelly 2011).

Mussels were fed every second day with a commercially produced phytoplankton mix (Reed Mariculture, Shellfish Diet-1800 Formula, Campbell, CA, USA) following a water colour monitoring technique recommended by Reed Mariculture; Enough algae were added to require the mussels ~75 minutes to clear the water. Mussels additionally feed on phytoplankton naturally occurring in the water.

### Experimental design

A temperature ramp experiment was applied to determine survival after exposure to realistic high and sub-zero air temperatures (Thyrring *et al*., 2015). Mussels were exposed to seven high (24; 27; 32; 33; 37; 38; 41°C) and seven sub-zero (−3; −5; −7; −9; −11; −13; −15°C) air temperatures. Fifteen mussels from each collection site were used at every temperature (a total of 420 mussels). Mussels were removed from the holding aquarium and, placed on a tray, moved to a climate chamber.

In the field, mussel body temperatures gradually change doing submergence/emergence. To avoid unwanted initial heat-shock responses from transferring mussels from 10°C water temperature to e.g. 41°C warm air, we gradually increased air temperatures to get more realistic heat-shock response (Thyrring *et al*., 2015). To do this, we used a ramp air temperature trial experiment with a constant gradual temperate change over 45 minutes. This change simulates the period of low tide when mussels are gradually exposed to air. Mussels were kept at a constant experimental temperature (temperature varied within ±1°C of the target temperature) for 60 minutes, which equals exposure time at the collection sites, before a reversed 45 minutes change of air temperature to 10°C was initiated. During the 45 minutes, temperatures changed at a rate varying from 0.31 to 0.68°C min^−1^, depending on the final experiment temperature. We used a variable, rather than a constant, temperature rate change because this is an important component of organismal thermal stress. For example, intertidal organisms experience more rapid temperature changes on days with extreme temperatures. Following aerial exposure, each mussel was transplanted to separate containers and submerged in a flow through aquarium (10°C). Survival was evaluated after 1 hour because previous experiments found no differences in mortality when evaluated 1 hour and 24 hours following air exposure (Thyrring *et al*., 2015). Mussels were considered dead if they did not close their shells when touched or prodded while still submerged in water (following Peck *et al*., 2009). It should be noted that the sub-zero temperatures we used are low enough to cause freezing of blue mussel body fluids (Aarset and Zachariassen 1982; Thyrring *et al*., 2015). Surviving mussels were flash frozen to −80°C and stored at −80°C for fatty acid analysis.

We estimated the upper and lower lethal temperature (LT_min_ and LT_max_) where all individuals died, and the temperature where 50% of individuals died (LT_min50_ and LT_max50_). Thermal window was calculated as the difference between LT_max_ and LT_min_.

### Fatty acid analysis

Fatty acid analyses were performed on gill tissue (average sample wet weight = 0.19 ± 0.12 g, *n* = 3–5, depending on number of survivors [for full dataset see Thyrring *et al*., 2019]). We chose to study gills because this tissue is important for bivalves (e.g. oxygen consumption, food sorting and ingestion) and the lipid composition of this tissue responds readily to acclimation temperature, as predicted by homeoviscous adaptation, in oysters and mussels (Pernet *et al*. 2007b; Thyrring *et al*. 2017c).

Total lipids were extracted by grinding in dichloromethane:methanol (2:1, v:v) solution following a slight modified Folch procedure described in Parrish (1999). Lipid extracts were separated into neutral and polar fractions by column chromatography on silica gel micro-columns (30 × 5 mm i.d., packed with Kieselgel 60, 70–230 mesh; Merck, Darmstadt, Germany) using chloroform:methanol (98:2, v/v) to elute neutral lipids, followed by methanol to elute polar lipids (Marty *et al*., 1992). Fatty acid profiles on polar lipids were determined on fatty acid methyl esters (FAMEs) using sulphuric acid:methanol (2:98, v:v) and toluene. FAMEs of neutral and polar fractions were concentrated in hexane and the neutral fraction was purified on an activated silica gel with 1 mL of hexane:ethyl acetate (v/v) to eliminate free sterols. FAMEs were analysed in the full scan mode (ionic range: 50–650 m/z) on a Polaris Q ion trap coupled multichannel gas chromatograph “Trace GC ultra” (Thermo Scientific, MA, USA) equipped with an autosampler model Triplus, a PTV injector and a mass detector model ITQ900 (Thermo Scientific, MA, USA). The separation was performed with an Omegawax 250 (Supelco) capillary column with high purity helium as a carrier gas. Data were treated using Xcalibur v.2.1 software (Thermo Scientific, MA, USA). Methyl nondecanoate (19:0) was used as an internal standard. FAMEs were identified and quantified using known standards (Supelco 37 Component FAME Mix and menhaden oil; Supleco), and were further confirmed by mass spectrometry (Xcalibur v.2.1 software). In all samples, unknown peaks were identified according to their mass spectra with emphasis on fatty acid trophic markers.

### Statistical analysis

Shell size is known to affect freeze tolerance of intertidal mussels (Aarset, 1982; Thyrring *et al*., 2017a). Prior to analysis, we therefore tested if shell length differed amongst temperatures and locations using two-way analysis of variance (ANOVA). The test showed that shell sizes was statistically similar between temperatures (*F*_13,406_ = 0.95, *P* = 0.497) and amongst locations (*F*_1,418_ = 0.008, *P* = 0.931). Based on these results, we analysed differences in survival using a generalised linear model (GLM) with a binomial distribution (McCullagh and Nelder, 1989).

We found 18 fatty acids that each contributed >0.5% of the total amount. We analysed this data with a distance-based permutational multivariate analysis of variance PERMANOVA (9999 permutations) after verification of assumptions of homoscedasticity with a PERMDISP. The fatty acids explaining most of the dissimilarity between the Arctic and temperate population was identified using similarity percentage analysis (SIMPER). SIMPER use the contribution of each FA to calculate the average dissimilarity (or similarity) between temperature treatments and locations, and then assesses the relative dissimilarity contributed by each fatty acid. SIMPER uses Bray-Curtis measure of similarity (Bray and Curtis, 1957). This analysis revealed that seven fatty acids explained ~80% of the Bray–Curtis dissimilarity amongst fatty acid profiles in the two populations and between temperatures. We therefore focused all subsequent analysis on these seven fatty acids.

ANOVAs were used to evaluate differences in the amount of saturated fatty acids (SFA; fatty acids with no double carbon bonds), monounsaturated fatty acids (MUFA; fatty acids with a one double carbon bond), polyunsaturated fatty acids (PUFA; fatty acid chains containing more than one double carbon bond), unsaturation index (UI; a measure of the number of double bonds calculated as the sum of the percentage of each unsaturated fatty acid multiplied by the number of double bonds within the fatty acid) amongst locations and temperatures. TukeyHSD *post hoc* pair-wise tests were used to compare significant treatment effects (*P* < 0.05). Detailed data exploration was carried out prior to any analysis. SFA, PUFA, MUFA and UI data showed homoscedasticity and normality of distribution. Once valid models were identified, we re-examined the residuals to ensure model assumptions were acceptable.

## Results

### Air temperatures

Air temperatures were measured at seven weather stations (Fig. 1a). The mean annual temperature across years decreased with latitude from 10.1 to −8.8°C (Fig. 1b), with minimum and fifth percentile temperatures decreasing in a similar pattern (Fig. 1c and d). The minimum temperature decreased from −22 to −40.5°C, and the fifth percentile from −1 to −27.1°C, from location 1 to 7, respectively. However, maximum and 95th percentile temperatures exhibited a substantially different pattern. The highest temperature measured, markedly decreased from the temperate (location 1) to the subarctic region (location 2), but the variance along the west Greenland coast (locations 2–7) was less than 3°C (95th percentile) and 8°C (maximum) (Fig. 1c and d). Consequently, the local annual air temperature range increased with latitude from a minimum of 37.5 (location 2) to 60.8°C (location 7) (Fig. 1e).

The fifth percentile near the two sampling sites were − 1°C in Denmark and −12.8°C in Greenland.

### Survival of blue mussels

**Figure 2:**
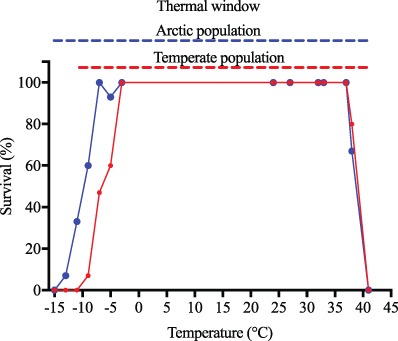
Survival (%) after 60 minutes air exposure to various air temperatures in *M. edulis* from the Arctic (blue) and temperate region (red), *n* = 15 for each experimental temperature. Thermal windows are indicated as horizontal dotted lines.

There was a significant difference in the lower thermal limit of *M. edulis* from the Arctic and temperate region (GLM; ${\chi}^2=163.7$, df = 6, *P* < 0.0001, Fig. 2). Individuals from the Arctic population had a significantly lower LT_min50_ (= −10.55°C) than their conspecifics from Denmark (LT_min50_ = −6.25°C). The lower LT was 4°C lower in the Arctic (LT = −15°C) compared to the temperate region where no mussels survived temperatures of −11°C (Fig. 2). There were no significant differences in upper LT (>38°C) and LT_max50_ amongst Arctic (LT_max50_ = 38.03°C) and temperate (LT_max50_ = 38.06°C) populations (Fig. 2). The thermal window of Arctic mussels was 53°C compared to 49°C of temperate mussels.

### Fatty acids

There was a significant difference between Arctic and temperate mussels in the proportion of the fatty acids 20:1ω9 and 20:5ω3 (Table 1), but no differences in SFA, MUFA, PUFA or UI was found (Table 1). Air temperatures had a significant effect on the fatty acids 17:1, 20:1ω9 and 22:6ω3 in temperate mussels (Table 2). In Arctic mussels, temperatures significantly affected the amount of 17:1, 20:1:ω9, 20:5ω3 and 22:6ω3, and MUFA, PUFA and UI (Table 2).

**Table 1:** Mean percentage (± standard deviation) distribution of seven fatty acids in surviving Arctic and temperate mussels. The summarised degree of SFA, MUFA, PUFA and UI is presented. The table presents the seven fatty acids, which contributed ~ 80% of the SIMPER dissimilarity amongst the two locations. Bold *F*-values, degrees of freedom (df) and *P*-values indicate significant differences in fatty acids between locations

Fatty acids	Temperate	Arctic	*F*-value	df	*P*-value
16:0	5.87 ± 2.1	5.59 ± 2.6	0.33	1	0.570
17:1	22.25 ± 11.4	20.29 ± 8.8	0.34	1	0.337
20:1ω9	9.38 ± 1.8	12.87 ± 1.9	**85.57**	**1**	**<0.0001**
20:4ω6	8.70 ± 1.9	9.08 ± 1.8	0.98	1	0.324
20:5ω3	11.63 ± 3.3	8.86 ± 2.3	**24.41**	**1**	**<0.0001**
22:2	13.66 ± 2.3	13.57 ± 2.3	0.03	1	0.854
22:6ω3	14.16 ± 3.9	13.61 ± 3.6	0.53	1	0.578
SFA	10.86 ± 3.4	10.96 ± 4.5	0.02	1	0.897
MUFA	37.40 ± 10.5	39.32 ± 7.9	1.05	1	0.308
PUFA	51.74 ± 9.1	49.72 ± 7.2	1.52	1	0.221
UI	252.19 ± 33.1	240.85 ± 27.8	3.41	1	0.068

**Table 2:** Mean percentage (± standard deviation) distribution of fatty acids in surviving Arctic and temperate mussels exposure to various air temperatures. The summarised degree of SFA, MUFA, PUFA and UI is presented. Bold *F*-values, df and *P-*values indicate significant effect of temperature on fatty acid composition.

Fatty acids	−11	−9	−7	−5	−3	24	27	32	33	37	38	*F*-value	df	*P*-value
**Arctic**														
16:0	5.3 ± 2.8	7.5 ± 6.1	7.4 ± 2.2	4.6 ± 0.9	5.6 ± 1.4	5.9 ± 2.1	7.3 ± 3.2	6.8 ± 1.2	3.7 ± 1.0	4.6 ± 0.8	3.1 ± 0.7	2.97	10	0.066
17:1	20.9 ± 7.4	18.9 ± 11.8	16.7 ± 6.4	14.8 ± 12.2	14.3 ± 13.0	22.3 ± 2.5	22.5 ± 7.7	14.0 ± 7.6	26.5 ± 5.7	23.6 ± 4.8	28.4 ± 4.7	**1.81**	**10**	0.088
20:1ω9	12.5 ± 1.3	13.7 ± 1.7	14.5 ± 1.3	14.0 ± 2.1	13.3 ± 2.5	11.4 ± 1.8	13.3 ± 2.5	12.0 ± 1.9	12.5 ± 1.8	13.1 ± 1.2	11.3 ± 0.8	1.65	10	**0.013**
20:4ω6	10.7 ± 1.9	7.6 ± 2.0	9.6 ± 0.9	9.8 ± 2.3	9.8 ± 1.8	8.4 ± 1.8	8.9 ± 2.2	9.5 ± 1.2	8.4 ± 1.5	9.1 ± 2.0	7.8 ± 1.4	1.34	10	0.243
20:5ω3	9.4 ± 1.4	9.9 ± 1.0	7.6 ± 1.0	11.2 ± 2.5	10.1 ± 1.4	7.9 ± 1.0	7.3 ± 1.2	11.5 ± 4.0	6.0 ± 1.1	8.4 ± 1.0	8.3 ± 1.0	**4.71**	**10**	**0.0002**
22:2	12.2 ± 2.3	14.5 ± 3.3	12.7 ± 2.4	14.3 ± 3.1	14.5 ± 3.2	12.6 ± 2.0	13.3 ± 2.2	13.7 ± 2.9	14.1 ± 0.6	14.1 ± 1.0	13.6 ± 1.9	0.57	10	0.83
22:6ω3	12.1 ± 3.1	14.0 ± 3.1	13.6 ± 2.8	16.0 ± 3.0	17.8 ± 5.0	12.0 ± 3.0	10.9 ± 1.2	17.8 ± 3.7	12.3 ± 1.5	11.6 ± 2.2	11.9 ± 2.4	**3.52**	**10**	**0.002**
SFA	11.5 ± 7.6	13.1 ± 8.4	14.2 ± 3.8	9.5 ± 1.3	10.5 ± 2.2	13.0 ± 5.2	13.4 ± 5.3	12.1 ± 1.7	7.7 ± 1.6	9.1 ± 1.7	6.9 ± 1.1	1.59	10	0.144
MUFA	40.2 ± 5.8	37.3 ± 10.8	38.1 ± 6.6	34.8 ± 9.7	32.7 ± 10.1	40.7 ± 2.4	41.2 ± 5.5	31.3 ± 8.2	46.0 ± 4.2	43.1 ± 4.5	46.6 ± 4.5	**2.59**	**10**	**0.015**
PUFA	48.3 ± 6.5	49.7 ± 4.8	47.7 ± 6.0	55.7 ± 9.3	56.8 ± 8.3	46.3 ± 6.3	45.3 ± 5.1	56.6 ± 7.4	46.3 ± 3.0	47.8 ± 6.0	46.5 ± 4.0	**2.44**	**10**	**0.021**
UI	237.2 ± 26.6	239.8 ± 12.8	232.6 ± 19.4	265.6 ± 27.5	269.6 ± 27.3	225.4 ± 26.4	218.7 ± 18.2	271.6 ± 33.1	226.0 ± 10.7	231.8 ± 22.1	230.8 ± 16.3	**3.39**	**10**	**0.002**
**Temperate**														
16:0	–	–	6.0 ± 1.8	7.6 ± 2.5	6.0 ± 3.1	6.6 ± 1.9	7.0 ± 2.4	6.0 ± 1.5	4.9 ± 1.6	4.1 ± 1.5	5.0 ± 1.6	1.39	8	0.236
17:1	–	–	11.9 ± 12.1	11.4 ± 13.9	23.2 ± 10.6	26.3 ± 6.5	23.3 ± 10.6	18.0 ± 13.3	24.4 ± 8.0	33.2 ± 2.0	28.7 ± 6.7	**2.69**	**8**	**0.020**
20:1ω9	–	–	10.9 ± 0.7	10.7 ± 2.1	8.7 ± 1.8	8.0 ± 0.9	9.8 ± 2.6	9.7 ± 2.3	8.9 ± 1.3	8.2 ± 0.5	9.6 ± 2.0	**1.79**	**8**	0.111
20:4ω6	–	–	10.0 ± 1.8	9.52.3	8.2 ± 1.7	8.1 ± 1.0	9.2 ± 1.9	9.2 ± 2.8	8.9 ± 2.1	7.6 ± 0.9	7.8 ± 2.0	0.87	8	0.549
20:5ω3	–	–	14.3 ± 4.0	14.0 ± 4.5	12.7 ± 3.5	11.4 ± 1.7	10.1 ± 2.6	12.3 ± 2.5	11.5 ± 2.7	9.5 ± 1.1	8.6 ± 2.2	2.12	8	0.06
22:2	–	–	15.8 ± 2.5	14.8 ± 4.1	12.8 ± 1.5	12.0 ± 1.6	14.3 ± 2.0	13.1 ± 1.2	14.2 ± 2.4	12.8 ± 1.9	13.2 ± 1.6	1.35	8	0.252
22:6ω3	–	–	17.2 ± 3.1	16.7 ± 5.4	16.0 ± 3.0	12.3 ± 2.1	11.6 ± 1.0	17.8 ± 4.0	13.9 ± 2.1	10.8 ± 1.0	10.8 ± 2.5	**4.29**	**8**	**0.001**
SFA	–	–	11.0 ± 2.7	14.3 ± 4.8	11.1 ± 4.2	11.6 ± 3.0	12.8 ± 3.7	10.8 ± 2.6	8.8 ± 2.2	8.1 ± 2.2	9.6 ± 2.0	1.83	8	0.104
MUFA	–	–	28.1 ± 11.2	27.4 ± 13.4	36.7 ± 9.6	41.0 ± 5.8	38.5 ± 7.8	33.1 ± 11.6	39.1 ± 8.2	47.5 ± 1.7	45.4 ± 5.4	2.99	8	0.012
PUFA	–	–	60.9 ± 9.1	58.3 ± 14.9	52.2 ± 5.5	47.4 ± 5.5	48.7 ± 4.1	56.1 ± 9.6	52.1 ± 7.3	44.4 ± 1.7	45.0 ± 5.2	2.67	8	0.021
UI	–	–	283.1 ± 30.8	273.2 ± 53.9	261.5 ± 21.8	237.7 ± 19.5	233.4 ± 7.9	273.8 ± 33.2	253.8 ± 24.2	226.5 ± 5.9	223.0 ± 18.9	3.17	8	0.008

## Discussion

﻿Understanding inter-population temperature sensitivity is fundamental to predict the impacts of climate change on a species distribution. However, while the importance of thermal tolerance and temperature adaptation in rear-edge populations are well studied (Willett, 2010; Bennett *et al*., 2019), much less is known about cold adaptation and its influence on distributional changes at the leading-edge. Our results demonstrate that the northernmost population of the blue mussel *M. edulis* exhibit a different absolute temperature tolerance compared to a temperate population. Specifically, following 5 months of acclimation under common garden conditions, Arctic blue mussels were able to survive sub-zero air temperature exposure down to −13°C, which is 4° lower than the thermal limit of the European population.

Physiological and biochemical adaptations to low temperatures are well known. For instance, having evolved in a constant cold environment, Antarctic benthos is highly stenothermal with a small thermal window and a poor capacity to acclimate to elevated temperatures (Peck *et al*., 2014). In temperate species, thermal adaptation involves change in enzymatic activity and gene expression, resulting in a reduced upper thermal tolerance in high latitude populations (Sokolova and Pörtner, 2001; King *et al*., 2018; Marshall *et al*., 2018). Our study demonstrates a different response in *M. edulis*. Arctic mussels survived 4° lower temperatures, yet maintained an upper thermal limit (~38°C) similar to that of temperate individuals, hereby increasing their thermal window. This adaptation could arise from either genetic local adaptation or non-genetic phenotypic plasticity (including maternal effects, modified morphology, physiological acclimatisation etc.; Piersma and Drent, 2003; Bayne, 2004; Wolf and Wade, 2009). There are two main reasons why we argue the observed increase in thermal window is caused by local adaptation. The first, and strongest, evidence for Arctic mussels being cold adapted is that a low gene flow across the Atlantic Ocean has resulted in a large and continuing genetic divergence amongst the two studied populations (Riginos *et al*., 2004; Mathiesen *et al*., 2017). In addition to geographic isolation, this trans-Atlantic split in genetic structure is likely driven by genetic adaptation to local environmental conditions (Mathiesen *et al*. 2017), facilitating the demonstrated increase in freeze tolerance. Secondly, all mussels were acclimated to common conditions for 5 months, which is adequate to minimise the confounding effects of environment history (Sokolova and Pörtner 2001), thus supporting local adaptation. However, determining actual local adaptation is difficult and requires raising several generations under common garden laboratory settings (Sanford and Kelly, 2011). Therefore, we cannot exclude that other processes, such as, early developmental acclimatisation or maternal effects, may be partly responsible for observed physiological variation.

The latitudinal downshift in freeze tolerance appears to occur without any trade-off in heat tolerance as both populations survive temperatures of 38°C. Temperatures impact organismal energetics and ultimately survival, and invariance in upper thermal limits throughout a species distribution range either represent a lack of selection pressure from current experienced temperatures (Clements *et al*., 2018; King *et al*., 2018), or results from different populations being seasonally exposed to similar high temperatures. Since the thermal window of the Arctic population correlate with the fifth percentile (−12.8°C), at the collection site, and Thyrring *et al*. (2017a) reported intertidal temperatures above 36°C near blue mussel beds in Greenland’s intertidal zone, we suggest Arctic mussels have increased their thermal window to cope with low and high-temperature stress. However, the impacts of high summer temperatures in the Arctic remain unknown.

### The importance of membrane fatty acid composition on freeze tolerance

The fatty acid composition of cell membranes affects the thermal tolerance of ectotherms (Hazel and Williams, 1990). For example, in response to low ambient temperatures, cold-adapted species decreases the degree of SFA to avoid phase transitions to a dysfunctional ordered gel phase (Hazel and Williams 1990; Hazel, 1995). Pernet *et al*. (2007a) demonstrated that the oyster *C. virginca* remodel membrane lipids following daily temperature fluctuations (12–25°C for 7 days), and compositional change in response to long-term adaptation and seasonal temperature changes occurs in terrestrial and aquatic organisms (Hazel, 1995; Thyrring *et al*., 2017c). However, we found no significant correlations between fatty acid composition and freeze tolerance following air exposure. Thus, although homeoviscous adaptation is important for maintaining cell functionality in response to short- and long-term water temperature changes, our results indicate limited importance of this process for surviving acute exposure to air in intertidal species. The physiological mechanisms for surviving low water temperatures might therefore be different from the processes ensuring survival to acute freezing. Inter-population differences in tolerance to freezing have been subscribed to differences in cellular concentrations of specific freeze-protective cryoprotectants. For example, increased haemolymph concentrations of calcium and taurine have together with anaerobic byproducts, such as strombine, been shown to protect membranes and increase freeze tolerance of intertidal bivalves (Murphy, 1977; Loomis *et al*., 1988). Furthermore, polar organisms often have additional heat shock proteins (HSP) (Peck, 2016), and the HSP production is upregulated in cold-adapted populations of intertidal barnacles (*Semibalanus balanoides*) following exposure to low air temperatures (Marshall *et al*., 2018). The role of HSP in freeze tolerance is poorly understood, yet these chaperones may protects cells from freeze induced cell destabilization and irreversible protein denaturation, and should be investigated further.

### Mosaic of interactions determine poleward distribution limits

Air temperatures are important for the distribution of intertidal species, and a central component in species distribution modelling (Elith and Leathwick 2009, Bennett *et al*. 2019). *M. edulis* collected in Denmark all died at air temperatures below −7 °C, putting the intertidal poleward limit of *M. edulis* would near the southern tip of Greenland. However, due to cold adaptation, *M. edulis* is found further north in Kobbefjord (Location 3, Fig. 1a) and, since *M. edulis* inhabit intertidal microhabitats that reduce exposure to extreme temperatures (Thyrring *et al*., 2017a), individuals are found as north as 71°N in Greenland (location 6, Fig.), (Mathiesen *et al*., 2017). Our study highlights the importance of including local cold adaptation (and phenotypic plasticity) in the effort to understand climate-change-induced range shifts.

Complex interactions involving several biological and environmental factors are important for shaping species thermal tolerance and distribution. For instance, behaviour is an important trait for the survival of mobile species as they move between microhabitats that provide thermal refuge (Dong *et al*., 2017). Shell-forming organisms adjust shell morphology in response to environmental conditions (Telesca *et al*., 2018) and, further, can other variables such as, salinity, pH, and food availability affect species distribution by modifying thermal tolerance limits (Gunderson *et al*., 2016). Thus, understanding the combined effects of these interactions is important to predict distributional patterns.

Therefore, instead of readily applying uncertain correlative distribution models to various organisms from across the globe, we suggest using mechanistic models focused on key species with wide importance for ecosystems (such as, ecosystem engineers, foundation species or keystone species). Combining physiological adaption and plasticity with biotic (such as, competition, habitat use, dispersal and diseases) and environmental (such as, salinity, exposure time and pH) parameters, mechanistic modelling may start to provide highly realistic estimations from which climate change and conservation actions can be effectuated.

## Data Availability

The data for this study is available through Scholars Portal Dataverse repository and can be accessed at doi: 10.5683/SP2/HTDT55 (Thyrring *et al*., 2019).
